# Efficacy and Safety of Low-Dose Rivaroxaban in High-Ischemic-Risk Patients with Chronic Coronary Syndrome: Rationale and Design of the DUTCH CCS Registry

**DOI:** 10.3390/jcm14134401

**Published:** 2025-06-20

**Authors:** Abi Selvarajah, Dirk J. van der Heijden, Wouter S. Remkes, Jurriën M. ten Berg, Michael Magro, Clemens von Birgelen, Robert K. Riezebos, Ron Pisters, Martin E. W. Hemels, Saman Rasoul, Arnoud W. J. van ‘t Hof, Samer Somi, Jawed Polad, Pieter Hoogslag, Renicus S. Hermanides

**Affiliations:** 1 Department of Cardiology, Isala Hospital, 8025 AB Zwolle, The Netherlands; 2 Department of Cardiology, Haaglanden Medical Center, 2512 VA Den Haag, The Netherlands; 3 Department of Cardiology, VieCuri Medical Center, 5912 BL Venlo, The Netherlands; 4 Department of Cardiology, St. Antonius Hospital, 3435 CM Nieuwegein, The Netherlands; 5 Department of Cardiology, Elisabeth-TweeSteden Hospital, 5022 GC Tilburg, The Netherlands; 6 Department of Cardiology, Medisch Spectrum Twente, 7512 KZ Enschede, The Netherlands; 7 Department of Cardiology, OLVG Hospital, 1091 AC Amsterdam, The Netherlands; 8 Department of Cardiology, Rijnstate Arnhem Hospital, 6815 AD Arnhem, The Netherlands; 9 Department of Cardiology, Zuyderland Medisch Centrum, 6419 PC Heerlen, The Netherlands; 10 Department of Cardiology, Maastricht University Medical Center, 6229 HX Maastricht, The Netherlands; 11 Cardiovascular Research Institute Maastricht (CARIM), 6229 HX Maastricht, The Netherlands; 12 Department of Cardiology, Haga Hospital, 2545 CH Den Haag, The Netherlands; 13 Department of Cardiology, Jeroen Bosch Hospital, 5223 GZ Den Bosch, The Netherlands

**Keywords:** chronic coronary syndrome, low-dose rivaroxaban, dual-pathway inhibition

## Abstract

**Background/Objectives**: Despite progress in secondary prevention, people with chronic coronary syndrome (CCS) still face a residual risk of ischemic events. Antithrombotic therapy reduces this risk and helps stabilize chronic cardiovascular disease. Studies have shown that combining low-dose rivaroxaban with aspirin—an approach called dual-pathway inhibition (DPI)—can lower this risk and reduce major adverse cardiovascular events (MACEs). However, researchers have not yet gathered enough real-world data to confirm the efficacy and safety of this strategy. The DUTCH CCS registry aims to collect real-world data on how effective and safe low-dose rivaroxaban combined with aspirin is for patients with CCS in The Netherlands. The study aims to provide insights into the outcomes, benefits, and risks of DPI in a real-world setting, beyond the scope of controlled clinical trials. **Methods**: The DUTCH CCS registry operates as a national, multicenter, prospective observational study. It enrolls 1000 patients with CCS who receive rivaroxaban (2.5 mg twice daily) and aspirin (80 mg or 100 mg once daily). The study targets individuals at high ischemic risk due to coronary artery disease (CAD) and follows a single-arm design. Researchers will measure the primary efficacy endpoint by tracking MACEs, clinically driven coronary, peripheral, or carotid revascularization, and stent thrombosis over one year. They will assess the primary safety endpoint by recording major bleeding events at one year. The team will collect data at both 3-month and 1-year follow-ups. **Conclusions**: As an observational study, this registry is not designed to establish causality. However, it seeks to improve our understanding of how DPI performs in real-world secondary prevention for CCS patients. The results may help update treatment guidelines and inform clinical decisions in everyday practice.

## 1. Introduction

Chronic coronary syndrome (CCS) still poses a significant risk for major adverse cardiovascular events (MACEs). A patient’s clinical history—such as previous myocardial ischemia, obstructive coronary artery disease (CAD), and cardiovascular risk factors—strongly influences the incidence of these events. The REACH (Reduction of Atherothrombosis for Continued Health) registry reports 4-year MACE rates of 18.3% in patients with a history of ischemia, 12.2% in those with stable CAD, and 9.1% in patients with risk factors alone [1]. These data highlight the substantial residual risk in CCS and emphasize the need for continued vigilance and optimized therapy.

Clinicians typically use long-term single antiplatelet therapy with aspirin as the standard treatment for CCS. This approach reduces MACEs by about 22% annually. Despite this benefit, many patients still face a high residual ischemic risk, which calls for more refined antithrombotic strategies [2,3].

Researchers believe that persistent thrombin generation, which can last for months after an acute coronary event, contributes to this ongoing risk by driving inflammation and atherosclerosis [4,5,6]. In response, multiple studies have tested anticoagulants in both acute coronary syndrome and CCS patients for secondary prevention [7].

Although vitamin K antagonists lower ischemic events, they also raise the risk of major bleeding, which limits their usefulness. In contrast, rivaroxaban—a direct oral anticoagulant—offers a more favorable safety profile. It works by selectively inhibiting coagulation pathways, reducing thrombin production, and limiting platelet activation [8,9,10].

The COMPASS trial tested low-dose rivaroxaban in patients with stable CAD [11]. In the trial, patients received either rivaroxaban 2.5 mg twice daily with aspirin or rivaroxaban 5 mg once daily alone. The combination therapy lowered the risk of MACEs by 24% compared to aspirin alone, mainly by reducing strokes. Rivaroxaban alone did not show additional benefit and had a similar bleeding profile to aspirin. Among high-risk patients—including those with multivessel CAD, diabetes, prior myocardial infarction (MI), peripheral artery disease (PAD), heart failure, or moderate kidney disease—the combination therapy led to even greater absolute risk reduction [12]. However, it also caused more major bleeding (3.1% vs. 1.9%).

The VOYAGER-PAD trial produced similar findings. In patients with PAD who underwent revascularization, rivaroxaban combined with aspirin reduced MACEs more effectively than aspirin alone [13].

Based on this evidence, the European Society of Cardiology guidelines recommend low-dose rivaroxaban for CCS patients who have completed twelve months of dual antiplatelet therapy (DAPT) without major bleeding and have no history of stroke or transient ischemic attack. This recommendation holds a Class IIa–IIb, Level A rating [14,15]. Clinicians should especially consider this strategy for patients with high thrombotic risk and acceptable bleeding risk. They may also apply it to patients with moderate thrombotic risk.

Although clinical trials have shown promising results, researchers still lack sufficient real-world data on low-dose rivaroxaban in high-risk CCS patients. To address this gap, the DUTCH CCS registry has been established. Moreover, randomized trials often exclude elderly, frail, or multi-morbid patients. This results in a paucity of real-world evidence on DPI’s net clinical benefit across broader populations. Our registry addresses this gap by evaluating DPI in a pragmatic clinical environment, with broader inclusion criteria reflective of contemporary cardiology practice.

This observational study focuses on Dutch patients with CCS who face a high risk of ischemic events but have a relatively low bleeding risk. The study evaluates the safety and effectiveness of DPI in routine clinical settings. We hypothesize that DPI improves clinical outcomes without significantly increasing bleeding, even under real-world conditions. Because the study includes a broader high-risk population and defines outcomes more broadly—including revascularization and stent thrombosis in addition to MACEs—we expect to observe a higher rate of ischemic events than reported in randomized trials.

## 2. Methods

### 2.1. Study Design

**Figure 1:**
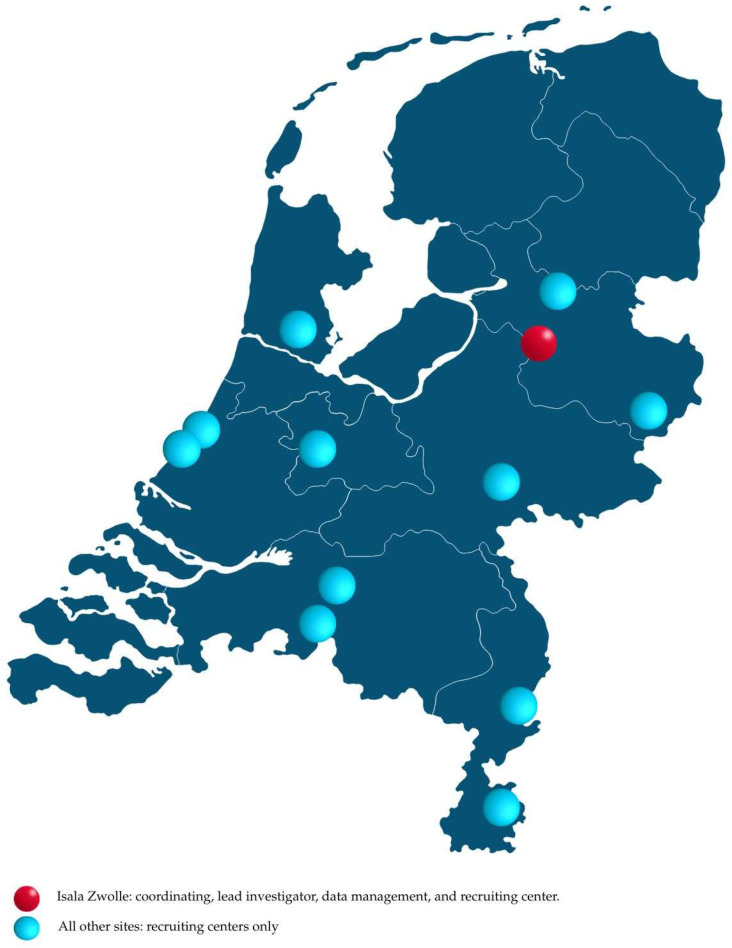
Geographic location and specific roles of the participating centers in The Netherlands.

The DUTCH CCS registry is a nationwide, multicenter, prospective, observational, single-arm study involving 1000 CCS patients who are treated with rivaroxaban 2.5 mg twice daily in addition to aspirin 80–100 mg once daily. The study is conducted in twelve interventional cardiac centers, situated across various regions of The Netherlands. Investigators at Isala Hospital in Zwolle are responsible for the oversight and coordination of the registry and data management. Figure 1 presents the geographic locations and specific roles of the twelve participating study sites.

From December 2020 to May 2022, this registry has been enrolling consecutive adult patients (aged 18 years or older) who are diagnosed with CAD, exhibiting one or more of the following high-ischemic-risk features: (1) coexistence of PAD, (2) recurrent MI (subsequent to an initial MI), (3) diabetes mellitus, (4) chronic kidney disease (eGFR 30–59 mL/min/1.73 m^3^), (5) heart failure (with an ejection fraction ranging from ≥30% to 50%) and New York Heart Association (NYHA) class I or II, and/or (6) CHA_2_DS_2_-VASc score (greater than 3 for men or greater than 4 for women). Although patient recruitment was completed between December 2020 and May 2022, the study is designed as a prospective observational registry from the outset.

All data collection and follow-up procedures are conducted in real time, in accordance with a predefined protocol established prior to enrollment. While the current manuscript is written after data collection, the analysis is based on prospectively gathered data, consistent with the study’s original design and intention.

CAD is characterized by the presence of one or more of the following conditions: (1) a history of previous MI, (2) stable angina or unstable angina with documented multi-vessel CAD, evidenced by over 50% stenosis in at least two major coronary arteries as revealed by coronary angiography, or positive stress test results (either electrocardiogram or nuclear perfusion test), (3) prior multivessel percutaneous coronary intervention, or (4) multivessel coronary artery bypass grafting surgery (CABG) performed within the past week or at least four years ago, or with recurrent angina or ischemia at any time following surgery. The rationale for this classification is based on the understanding that the risk of thrombotic events and graft failure is elevated during the initial year post-CABG. Subsequently, after a relatively stable period marked by low event rates between years 1 and 4, the thrombotic event risk starts to increase again.

PAD is characterized by (1) a history of prior aorto-femoral bypass surgery, limb bypass surgery, or percutaneous transluminal angioplasty involving iliac or infra-inguinal arteries, (2) previous limb or foot amputation specifically due to arterial vascular disease (excluding cases resulting from trauma), or (3) a documented history of intermittent claudication coupled with either an ankle/arm blood pressure ratio ≤ 0.90 or clear evidence of significant peripheral artery stenosis (>50%), confirmed by angiography or non-invasive testing using duplex ultrasound. A comprehensive outline of the inclusion and exclusion criteria is presented in Table 1.

Enrollment in the study relies on the treating physician’s decision to prescribe rivaroxaban alongside aspirin, based on medical indication. The decision to initiate DPI is independent of a patient’s inclusion in the study. Following this, patients on DPI are invited to participate in the DUTCH CCS registry. This approach aims to include patients previously on aspirin alone, for whom combination therapy with low-dose rivaroxaban is deemed appropriate due to their high-ischemic-risk profile. Patients already on DAPT, monotherapy with a P2Y_12_ inhibitor, or oral anticoagulation for another indication are not eligible. Moreover, patients are enrolled consecutively to ensure the study sample is representative of the CCS population.

### 2.2. Ethics and Informed Consent

Ethical approval for the study was granted by the non-WMO (WMO = Medical Scientific Research Act) Advisory Committee of a recognized Ethics Committee on behalf of the DCRF (‘Dutch Clinical Research Foundation’) for all sites after reviewing the protocol, site-specific informed consent forms, and other requested documents. Under Dutch law, this classification applies to research that does not involve interventions or procedures beyond standard clinical practice. As our study is observational and does not involve additional risk or burden to participants, formal review by a medical ethics committee under the WMO was not required.

Before participation, the treating physician informs the patient orally and in writing about the scope and purpose, rights, duties, and possible risks/benefits of the registry in lay language. Written informed consent from the patient is a prerequisite to participate in the registry. To maintain confidentiality, patients are identified only by their identification code. All patients will also receive a patient information form.

All study-related information will be stored securely at the study site. All participant information will be stored in locked file cabinets in areas with limited access. All reports, collected data, and process and administrative forms will be identified by a coded identification number only to maintain participant confidentiality. All records that contain names or other personal identifiers, such as locator forms and informed consent forms, will be stored separately from study records, identified by code number. All local databases will be secured with password-protected access systems. The study findings will be disseminated via publication of peer-reviewed manuscripts.

The results will be published irrespective of whether the findings are positive or negative.

### 2.3. Study Population

Patients, both women and men, diagnosed with CAD enroll in the outpatient clinic following the treating physician’s decision to initiate treatment with rivaroxaban 2.5 mg twice daily, in combination with aspirin 80–100 mg once daily. Inclusion criteria apply to patients visiting the outpatient clinic from Q4 2020 to Q2 2022 who meet the specified requirements and provide written informed consent. The study diligently considers indications and contraindications in accordance with the local market authorization.

In alignment with the study’s objective to capture real-life data, the inclusion and exclusion criteria are less stringent compared to randomized settings (e.g., the COMPASS trial). This approach reflects the considerable diversity of our patients and the varying complexity of the medical cases encountered in everyday clinical practice.

### 2.4. Study Size

The DUTCH CCS registry is a prospective, observational, single-arm study designed for descriptive and exploratory analysis. The study aims to enroll 1000 patients with chronic coronary syndrome (CCS), treated with low-dose rivaroxaban in addition to aspirin. The target sample size reflects a balance between feasibility and the goal of generating clinically meaningful insights within the study’s timeframe. Enrollment will continue beyond 1000 patients if needed to compensate for a dropout rate exceeding 5%, ensuring that sufficient patients are available for 12-month follow-up.

Although no formal power calculation was performed, the sample size is expected to provide adequate precision to estimate event rates for major outcomes, including MACEs and major bleeding, with 95% confidence intervals. Expected event rates were informed by incidence proportions observed in the COMPASS trial. However, due to the shorter follow-up period in our study (12 months vs. 23 months in COMPASS), the incidence of similar endpoints is expected to be lower. Conversely, the broader definition of clinical outcomes in our registry—which includes not only conventional MACEs but also clinically indicated coronary, peripheral, or carotid revascularization and stent thrombosis—may lead to higher overall event rates.

Furthermore, although the patient population largely resembles that of COMPASS, we include patients with higher CHA_2_DS_2_-VASc scores, potentially increasing the observed number of ischemic events. Based on these factors, we estimate that most clinical endpoints will occur in 1% to 5% of patients, which allows for reasonably precise estimates of outcome frequencies in relevant subgroups.

To support interpretation and assess the robustness of our findings, we will conduct sensitivity analyses. These include stratified analyses by clinical risk factors (such as diabetes, PAD, or prior MI), comparisons based on narrow versus broader endpoint definitions, evaluation of patients with complete versus incomplete follow-up, and exploration of site-level variability. These analyses will contextualize the observed outcomes and help determine the consistency of results across different analytical assumptions, despite the absence of formal hypothesis testing.

### 2.5. Study Endpoints

The primary effectiveness endpoint is a composite of the following events at one year:

- Major adverse cardiac events (MACEs; composite of cardiovascular death, myocardial infarction (MI), or stroke);
- Clinically driven coronary, peripheral, or carotid revascularization;
- Stent thrombosis.

Clinically driven revascularization is defined as a de novo hospital admission that leads to revascularization (excluding planned or staged procedures). Definite or probable stent thrombosis is defined according to the Academic Research Consortium (ARC) criteria [16]. Myocardial infarction is defined according to the fourth universal MI criteria [17].

The primary safety endpoint is major bleeding during one year. These major bleeding complications are analyzed according to the International Society on Thrombosis and Haemostasis (ISTH) criteria as a composite of (1) fatal bleeding, (2) symptomatic bleeding into a critical organ (such as intracranial, intraspinal, intraocular, retroperitoneal, intra-articular or pericardial, or intramuscular with compartment syndrome), or (3) bleeding causing a fall in hemoglobin level of 2 g/dL (1.24 mmol/L) or more or leading to transfusion of two or more units of whole blood or red cells [18]. In addition, all bleeding events, including minor bleedings according to ISTH definitions, will be reported.

Secondary endpoints will be the following:

- Occurrence (and date) of stroke.
- Occurrence (and date) of MI.
- Occurrence (and date) of cardiovascular death.
- Occurrence (and date) of coronary revascularization procedures (PCI, CABG).
- Occurrence (and date) of peripheral revascularization procedures.
- Occurrence (and date) of carotid revascularization procedures.
- Occurrence (and date) of minor bleeding complications according to ISTH (clinically relevant bleeding that does not meet criteria for major bleeding and that requires any medical or surgical intervention to treat the bleeding).

A detailed overview of the primary endpoints is provided in Table 2.

### 2.6. Data Collection and Follow-Up

The members of the study team prospectively gather data. During the baseline visit (T = 0), they collect historical data (demographic, laboratory—including parameters like hemoglobin, eGFR, and liver function—and clinical characteristics, including medication usage) from available medical records or through patient interviews if records are unavailable.

Primary and secondary endpoints are recorded at both 3-month and 1-year follow-up. The 3-month follow-up is conducted via telephone interview. Laboratory results are noted to the extent available within routine clinical practice, without performing additional study-related laboratory analyses. Patients are queried about adverse events, side effects of rivaroxaban, and any changes in their antithrombotic regimen (e.g., temporary cessation of aspirin, rivaroxaban, or both) and other medications. The time of final data collection is at one year, either through a face-to-face visit or via telephone contact (±30 days), or at 30 days in case of an early discontinuation of rivaroxaban therapy.

Potential reasons for premature study termination include consent withdrawal, loss to follow-up, or death of the patient. At this final observation, the patient’s condition and treatment assessment will be documented.

The treating physician determines the actual duration of treatment, which is not contingent on the initially intended treatment duration. In patients who permanently discontinue the combined treatment, discontinue rivaroxaban alone, or discontinue aspirin alone and who do not withdraw consent or experience a fatal adverse event during the observational period, the treating physician collects information on their survival status at the end of the study (i.e., 1-year ± 30 days after study inclusion).

A more detailed description of the variables collected during these visits is found in Table 3.

### 2.7. Data Management and Source Verification

The investigator or designated authorized site personnel will enter all clinical data anonymously in an electronic case report from (e-CRF) called eDREAM (Diagram BV, Zwolle, The Netherlands; version 1.0). This e-CRF is a secure, password-protected database that is backed up frequently. The structure of the CRF is available upon request.

The clinical research organization (CRO) performs data source verification through monitoring procedures. Physical monitoring visits were performed to ensure that written informed consent, adverse vents, and data accuracy were properly documented and available. This approach was selected to balance effective monitoring with feasibility and safety considerations.

### 2.8. Data Analysis

All statistical analyses were performed using IBM SPSS Statistics, version 28.0 (IBM Corp., Armonk, NY, USA).

The statistical analyses are explorative and descriptive. All analyses are performed for the total study population (overall analyses). The study period for analyses is defined as the time from the start of rivaroxaban until the end of the 1-year follow-up.

Descriptive statistics are performed for both primary and secondary study parameters. Continuous variables are expressed as mean ± standard deviation and as medians with minimum, maximum, 25th, and 75th percentiles. Categorical variables are presented by frequency tables (absolute and relative frequencies). Incidence proportions are computed together with corresponding exact 95% confidence intervals (CIs) for primary and secondary endpoints. Cumulative incidence of events or survival probabilities and 95% confidence intervals are estimated using Kaplan–Meier estimates for primary and secondary endpoints. The follow-up starts per definition on the date of the start of treatment and ends at the date of a first event, date of loss to follow-up, or date of end of the one-year study period, whichever comes first. The incidence of events over time is studied with the use of Kaplan–Meier methods. Patients who do not experience an event of interest by the end of their individual observation period are censored.

To control for confounding, the primary analyses will employ multivariable Cox proportional hazards regression models, with adjustment for prespecified baseline covariates known to influence clinical outcomes: age, sex, presence of diabetes mellitus, renal function, and CHA_2_DS_2_-VASc score. Model assumptions, including proportional hazards, will be assessed using Schoenfeld residuals. In addition, propensity score methods will be implemented as sensitivity analyses to further address confounding and potential selection bias. Propensity scores will be estimated using logistic regression models including the aforementioned covariates. These scores will be incorporated via adjustment for the propensity score as a covariate in the Cox model.

Missing data will be categorized by cause (e.g., treatment discontinuation, withdrawal of consent, or loss to follow-up) and described in detail. For time-to-event analyses (e.g., Kaplan–Meier estimation), patients will be right-censored at the date of last contact if no event has occurred. Dropouts due to reasons unrelated to outcome (e.g., relocation or administrative issues) will be assumed non-informative.

For other outcome measures, complete-case analysis will be the primary analytic approach. In addition, we will perform sensitivity analyses using multiple imputation under the assumption of missing at random, where appropriate. This approach allows us to explore the robustness of our findings and assess potential bias due to missing data. The dropout rate and its potential effect on external validity will be reported and discussed as part of the study limitations.

## 3. Trial Organization and Funding

The DUTCH CCS registry was registered on ClinicalTrials.gov with the registration number NCT04753372 on 15-02-2021. All data are collected in an electronic data capturing system, the electronic case record form, managed by Diagnostic Research and Management. Diagram BV, located in Zwolle, The Netherlands, is responsible for overall trial and data management, as well as for monitoring of the study. An independent physician of the clinical events committee (CEC) reviews and adjudicates all endpoint-related adverse events.

The Department of Cardiology of Isala Hospital, Zwolle, The Netherlands, sponsors the trial with funding from an unrestricted research grant from Bayer (Hoofddorp, The Netherlands). The primary investigators are solely responsible for the conduct of this registry, all study analyses, drafting and editing the manuscript, and its final contents. Diagram BV is the CRO responsible for performing this registry.

No patients or public are involved in the design of the study.

## 4. Discussion

The COMPASS [11] and VOYAGER-PAD [13] trials demonstrated significant benefits associated with DPI in patients with CCS and peripheral artery disease, respectively. Despite a limited number of randomized trials investigating low-dose rivaroxaban in CCS patients, the findings of the COMPASS trial motivated important updates to the current European Society of Cardiology guidelines for managing CCS. These guidelines now advocate for low-dose rivaroxaban in ACS patients who have successfully undergone twelve months of DAPT without bleeding complications. Specifically, the addition of low-dose rivaroxaban to aspirin should be considered for high-thrombotic-risk patients without an increased risk of major or life-threatening bleeding (Class IIa, Level A), and it may be considered for patients with moderately elevated thrombotic risk (Class IIb, Level A).

While real-world clinical practice often deviates from guideline recommendations, there is currently a dearth of real-world data about the potential impact of low-dose rivaroxaban on clinical outcome of CCS patients. Addressing this knowledge gap, the DUTCH CCS registry endeavors to provide valuable insights into the efficacy and safety of low-dose rivaroxaban in conjunction with aspirin. The focus of the study is on clinical practices and outcomes within a CCS patient population that is characterized by both a high thrombotic risk and a low bleeding risk.

Furthermore, although the majority of all ACS patients are treated by PCI, a substantial number of these patients is managed conservatively, representing a noteworthy portion of the entire CCS patient population. Conservative strategies are often employed for frail, elderly patients with multiple comorbidities, a demographic that is underrepresented in most of the randomized controlled trials. This is particularly true for women who are less prone to have obstructive CAD and might derive benefit from alternative treatment strategies as compared to men. Registry data from real-world clinical practice have the potential of providing signals that the use of low-dose rivaroxaban in combination with aspirin may be a viable alternative to single-antiplatelet therapy with aspirin alone.

Thus, the DUTCH CCS registry holds the potential to enhance our understanding of the real-world impact of low-dose rivaroxaban plus aspirin as a secondary prevention strategy in CCS patients with a high ischemic event risk and a low bleeding risk. The findings may offer valuable insights into the efficacy and safety of DPI, providing clinicians and researchers with data for informed treatment decisions, and may ultimately help refine guidelines for the management of CCS patients in routine clinical practice.

The strength of our study lies in its ability to represent patients encountered in daily clinical practice, utilizing broad inclusion criteria within a multicenter design. The COMPASS trial demonstrated that DPI significantly reduced MACE, major adverse limb events, and cardiovascular mortality in patients with stable CAD. Importantly, patients with diabetes mellitus, multivessel coronary disease and PAD derived the greatest absolute benefit, suggesting that DPI may be particularly effective in individuals with multiple high-risk features. These findings support the rationale for targeting patients with complex atherosclerotic disease who are at heightened thrombotic risk. In our registry, we included patients with PAD only when CAD was also present. This approach reflects current clinical understanding that the coexistence of CAD and PAD identifies a subgroup at especially high risk of ischemic events. We excluded patients with PAD in the absence of CAD, as their risk profiles and clinical management strategies may differ substantially, and they fall outside the focus of CCS as defined in our study. Our inclusion strategy aimed to better reflect the heterogeneity of patients encountered in routine clinical practice, as opposed to the narrow selection criteria used in randomized controlled trials. Data from the CLARIFY registry further justify this broader approach. CLARIFY showed that patients with higher CHA_2_DS_2_-VASc scores—even in the absence of atrial fibrillation—had increased long-term cardiovascular risk. By including patients with elevated CHA_2_DS_2_-VASc scores, our registry captures a wider range of real-world high-risk patients who may benefit from DPI but are often underrepresented in randomized controlled trials.

This pragmatic design allows the DUTCH CCS registry to evaluate the safety and effectiveness of DPI in a patient population that more closely mirrors everyday cardiology practice, improving the generalizability and clinical relevance of our findings.

However, there are several limitations inherent to the registry. One important limitation of this study is its single-arm, open-label design, which precludes direct comparison with a control group and limits causal inference. However, the decision to conduct the DUTCH CCS registry as a single-arm, observational study was deliberate, based on pragmatic and ethical considerations. Our objective was to capture real-world prescribing behavior and treatment outcomes in a high-risk CCS population eligible for DPI with rivaroxaban and aspirin. Importantly, the registry included prospectively defined inclusion and exclusion criteria, ensuring a well-characterized, guideline-eligible patient cohort rather than an unselected population. This structured approach enhances the internal validity of the findings while preserving external generalizability to routine clinical practice. While the absence of a comparator arm remains a limitation, it also avoids the artificial constraints and selection filters often imposed by randomized controlled trials. To address this limitation, future post hoc analyses using historical controls or propensity-score matching are under consideration. These methods may allow for contextualization of outcomes, provided that robust external datasets with compatible eligibility criteria and endpoints become available. At the time of study initiation, such data were lacking. Nevertheless, we believe that the DUTCH CCS registry provides valuable insights into DPI use across a broad and representative patient group, complementing evidence from randomized trials and helping to inform clinical decision-making in everyday practice.

Another potential limitation of this registry is the possibility of selection bias, as patients were enrolled based on predefined inclusion and exclusion criteria, but also at the discretion of the treating physician. However, it is important to clarify that the decision to initiate DPI was made independently of the registry, as part of routine clinical care. Patients were not prescribed DPI in order to participate in the registry; rather, only patients who had already been prescribed DPI based on clinical indication, and who fulfilled the inclusion criteria, were subsequently approached for participation. This approach reflects a real-world, pragmatic design aligned with the non-WMO (non–Medical Research Involving Human Subjects Act) classification of the study. To enhance representativeness and reduce potential selection bias, several quality assurance measures were implemented. All participating centers were instructed to include patients consecutively based on clear eligibility criteria. Additionally, retrospective verification of outpatient clinic records was performed at several sites to ensure that no eligible patients were unintentionally missed. Furthermore, audits and source data verification were conducted by an independent clinical research organization to assess adherence to inclusion criteria and to verify data quality. In cases where a patient was included but later found to be ineligible, the treatment plan was re-evaluated, and if DPI was deemed inappropriate, treatment was discontinued or the patient was excluded from the final analysis. While these procedures cannot entirely eliminate selection bias, they help to mitigate its extent and enhance the generalizability of our findings. Ultimately, our design aimed to capture real-world treatment patterns in a clearly defined, high-risk CCS population while maintaining scientific rigor within the practical and ethical constraints of an observational, non-interventional study. To further minimize potential inclusion bias, several centers conducted retrospective checks of their outpatient records to confirm that all eligible patients were considered for inclusion. Additionally, site monitoring and source data verification were performed by an independent clinical research organization to maintain data quality and adherence to inclusion criteria. To minimize inclusion bias and ensure comprehensive data collection, we will include all eligible patients according to pre-defined inclusion and exclusion criteria. Additionally, we will conduct a thorough review to confirm that no eligible patients were inadvertently excluded. Although the inclusion criteria might appear restrictive, as we specifically target high-risk patients with CCS, we believe this group is particularly relevant due to their persistent ischemic risk. Our objective is to provide real-world data, contrasting our findings with those of the COMPASS study. To ensure broad population representation and avoid underrepresentation of certain demographics or subgroups, we have selected centers from across the country.

A further limitation of this study is the relatively short follow-up duration of twelve months, which may not fully capture the long-term efficacy and safety outcomes of DPI in patients with CCS. Given the chronic and progressive nature of atherosclerotic cardiovascular disease, longer follow-up would be necessary to assess sustained ischemic protection, potential delayed bleeding risks, and treatment adherence over time. However, the 1-year timeframe was determined based on funding constraints imposed by the sponsor. Despite this limitation, we chose follow-up moments at 3 months and 1 year post DPI initiation to specifically monitor early adverse events—particularly bleeding—which typically emerge within the initial months of dual antithrombotic therapy. This design allows timely evaluation of tolerability and provides clinically relevant information on whether DPI can be safely continued or should be discontinued in real-world patients. Nonetheless, we recognize that late ischemic events and chronic safety concerns may not be fully captured. As such, an extended follow-up period is under consideration for future iterations of the registry, depending on feasibility and funding. This would enhance our understanding of the long-term net clinical benefit of DPI in this high-risk CCS population.

## 5. Conclusions

The DUTCH CCS registry is expected to provide valuable insights into the effectiveness and safety of low-dose rivaroxaban combined with aspirin in everyday clinical care for patients with chronic coronary syndrome. By collecting real-world data from a broad range of patients, the registry may help improve secondary prevention strategies and support future updates to treatment guidelines. Unlike randomized trials such as COMPASS or VOYAGER-PAD, the DUTCH CCS registry captures real-world prescribing behavior and treatment outcomes across a broader, more diverse population. The findings are expected to complement existing trial evidence by reflecting the effectiveness and safety of DPI in routine care. Moreover, these data could support refinement of risk stratification strategies and personalized implementation of DPI in ESC guidelines.

## Figures and Tables

**Table 1:** Overview of inclusion and exclusion criteria.

**Inclusion Criteria**
Adult patient (≥18 years) Diagnosis of CAD and high risk of ischemic events Patients at high risk of ischemic events include, amongst others, the following: ○ CAD + PAD ○ CAD + recurrent myocardial infarction (previous myocardial infarction followed by a second) ○ CAD + diabetes mellitus (all types) ○ CAD + chronic kidney disease (eGFR 30–59 mL/min/1.73 m^2^) ○ CAD + heart failure (ejection fraction ≥ 30–50%) and NYHA class I or II ○ CAD + high CHA_2_DS_2_-VASc score (men > 3 or women > 4) Patients can only be enrolled in the study if the decision to treat with rivaroxaban plus aspirin has been made by the treating physician in advance and independent of study inclusion within 4 weeks prior to study inclusion. Patients who are willing to participate in this study (signed informed consent)
**Exclusion Criteria**
Hypersensitivity/allergy and known contraindication to aspirin or rivaroxaban Patients with recent major bleeding, active bleeding, or one of the following: ○ History of major clinical bleeding or known coagulopathy ○ History of intracerebral mass, aneurysm, arteriovenous malformation, or hemorrhagic stroke Patients who received any organ transplant or await any organ transplant Patient with anemia (Hb < 6.0 mmol/L) Patient with active malignancy Patients with eGFR < 30 mL/min/1.73 m^2^ or undergoing dialysis Patients with liver failure accompanied with coagulopathy (Child-Pugh B and C) Patients with concomitant use of other anticoagulants or antiplatelet drugs Pregnant or lactating women Patients currently participating in another investigational drug or drug-coated device study

Abbreviations: CABG = Coronary Artery Bypass Grafting; CAD = Coronary Artery Disease; eGFR = Estimated Glomerular Filtration Rate; Hb = Hemoglobin; ISTH = International Society on Thrombosis and Haemostasis; MACE = Major Adverse Cardiovascular Event; PAD = Peripheral Artery Disease; PCI = Percutaneous Coronary Intervention.

**Table 2:** Primary endpoints.

Primary Efficacy Endpoints	Primary Safety Endpoints
Composite of the following events during one year: MACEs (composite of cardiovascular death, myocardial infarction or stroke) Clinically driven coronary, peripheral or carotid revascularization Stent thrombosis	Major bleeding according to ISTH during one year: Fatal bleeding Symptomatic bleeding into a critical organ Bleeding causing a fall in hemoglobin level of ≥2g/dL (1.24 mmol/L) or leading to transfusion of two or more units of whole blood or red cells.

Abbreviations: CABG = Coronary Artery Bypass Grafting; ISTH = International Society on Thrombosis and Haemostasis; MACEs = Major Adverse Cardiovascular Events; PCI = Percutaneous Coronary Intervention.

**Table 3:** Tabulated overview on study procedures and assessed variables.

Schedule Procedure	Initial Visit (Baseline)	Telephone Call/Report (3-Months FU)	Final Visit/Report at End of Observation (Last FU)
Date of informed consent	X		
Date of visit/report (day, month, year)	X	X	X
Demographic data	X		
Inclusion/exclusion	X		
Routinely collected laboratory data	X	X	X
Concomitant medication	X	X	X
Lifestyle	X		
Medical history	X		
Diagnosis of coronary artery disease	X		
Comorbidities	X		
Prior anticoagulation therapy *	X		
Assessment of antithrombotic therapy	X	X	X
Assessment of clinical outcomes		X	X
End of treatment *		X	X
Adverse events **		X	X

* If applicable. ** Adverse events (up to 30 days after the final treatment with rivaroxaban or until end of observation); (X) if applicable/available.

## Data Availability

The data underlying this study are available from the corresponding author upon reasonable request. Access to the data may be granted for research purposes, contingent on appropriate ethical approvals and data-sharing agreements to ensure participant confidentiality.

## Abbreviations

CABG	Coronary Artery Bypass Grafting
CAD	Coronary Artery Disease
CCS	Chronic Coronary Syndrome
DAPT	Dual Antiplatelet Therapy
DPI	Dual Pathway Inhibition
MACE	Major Adverse Cardiovascular Event
PAD	Peripheral Artery Disease
PCI	Percutaneous Coronary Intervention
